# Status of Onchocerciasis Transmission after More Than a Decade of Mass Drug Administration for Onchocerciasis and Lymphatic Filariasis Elimination in Central Nigeria: Challenges in Coordinating the Stop MDA Decision

**DOI:** 10.1371/journal.pntd.0003113

**Published:** 2014-09-18

**Authors:** Darin S. Evans, Kal Alphonsus, Jon Umaru, Abel Eigege, Emmanuel Miri, Hayward Mafuyai, Carlos Gonzales-Peralta, William Adamani, Elias Pede, Christopher Umbugadu, Yisa Saka, Bridget Okoeguale, Frank O. Richards

**Affiliations:** 1 The Carter Center, Atlanta, Georgia, United States of America; 2 The Carter Center Nigeria, Jos, Plateau, Nigeria; 3 University of Jos, Plateau, Nigeria; 4 Consultant, River Blindness Foundation, Atlanta, Georgia, United States of America; 5 Plateau State Ministry of Health, Jos, Plateau, Nigeria; 6 Nasarawa State Ministry of Health, Lafia, Nasarawa, Nigeria; 7 Department of Public Health, Federal Ministry of Health, Abuja, FCT, Nigeria; Noguchi Memorial Institute for Medical Research, Ghana

## Abstract

**Background:**

This study was undertaken in five onchocerciasis/lymphatic filariasis (LF) co-endemic local government areas (LGAs) in Plateau and Nasarawa, Nigeria. Annual MDA with ivermectin had been given for 17 years, 8 of which were in combination with albendazole. In 2008, assessments indicated that LF transmission was interrupted, but that the MDA had to continue due to the uncertain status of onchocerciasis transmission. Accordingly, assessments to determine if ivermectin MDA for onchocerciasis could be stopped were conducted in 2009.

**Methods:**

We evaluated nodule, microfilarial (mf) skin snip, and antibody (IgG4 response to OV16) prevalence in adults and children in six sentinel sites where baseline data from the 1990s were available. We applied the 2001 WHO criteria for elimination of onchocerciasis that defined transmission interruption as an infection rate of <0.1% in children (using both skin snip and OV16 antibody) and a rate of infective (L3) blackflies of <0.05%.

**Results:**

Among adult residents in sentinel sites, mean mf prevalence decreased by 99.37% from the 1991–1993 baseline of 42.95% (64/149) to 0.27% (2/739) in 2009 (p<0.001). The OV16 seropositivity of 3.52% (26/739) among this same group was over ten times the mf rate. No mf or nodules were detected in 4,451 children in sentinel sites and ‘spot check’ villages, allowing the exclusion of 0.1% infection rate with 95% confidence. Seven OV16 seropositives were detected, yielding a seroprevalence of 0.16% (0.32% upper 95%CI). No infections were detected in PCR testing of 1,568 *Simulium damnosum* s.l. flies obtained from capture sites around the six sentinel sites.

**Conclusion:**

Interruption of transmission of onchocerciasis in these five LGAs is highly likely, although the number of flies caught was insufficient to exclude 0.05% with 95% confidence (upper CI 0.23%). We suggest that ivermectin MDA could be stopped in these LGAs if similar results are seen in neighboring districts.

## Author Summary

Both lymphatic filariasis and onchocerciasis are treated with ivermectin-based mass drug administration (MDA) regimens in Africa. Where the infections are co-endemic, ivermectin treatments cannot be stopped until both infection transmission cycles are broken. This report follows a previous determination that the LF transmission cycle had been interrupted in five districts (LGAs in Nigeria) but evidence was needed on the status of the onchocerciasis transmission cycle prior to halting MDA. In this report we determined (based on WHO guidelines) that most likely the transmission of onchocerciasis has been interrupted in Plateau and Nasarawa States and we conclude that ivermectin MDA could be stopped.

## Introduction

Onchocerciasis is a leading cause of visual impairment and blindness in many developing countries. The main complications of this infection are severe eye disease that leads to blindness and skin disease characterized by papular or hypopigmented lesions and intense itching. The disease is caused by the filarial nematode *Onchocerca volvulus* that is transmitted by *Simulium* species black flies, the most common vector in sub-Saharan Africa being *S. damnosum* [1]. Fertilized female worms found in subcutaneous fibrous nodules, known as onchocercomas, release microfilariae (mf), the pre larval from of the parasite. These are picked up by the vector when blood feeding and eventually develop into the third stage larvae (L3) that are infectious to humans. The mf are also the cause of the major associated eye and skin morbidity of onchocerciasis. There are no epidemiologically important animal reservoirs [2].

Mass drug administration (MDA) with ivermectin is the WHO recommended strategy for the control of onchocerciasis [3]. Ivermectin is a potent microfilaricide that also has a limited effect on the viability and reproductive capabilities of adult onchocercal worms; females are able to regain their ability to produce microfilariae three to six months after ivermectin treatment [4]. This has meant that repeated treatment is needed in order to suppress the manifestations of the infection over time. Computer simulations have suggested that repeated ivermectin treatment could suppress transmission to a point at which the adult parasite population is no longer able to maintain itself, thereby interrupting transmission. While early models estimated this time at about 25 years [5], more recent evidence has suggested that 5 to 15 years of mass treatment with ivermectin could interrupt transmission depending on the treatment strategy and the initial force of infection [6], [7].

In Plateau and Nasarawa states in north-central Nigeria, onchocerciasis is endemic in 12 of the 30 districts (called in Nigeria Local Government Areas - LGAs) in the two states. Mass drug administration with ivermectin (Mectizan^R^, donated by Merck & Co., Inc., NJ, USA) in these 12 LGAs began in 1992 and has maintained >80% reported coverage of the treatment eligible population since 1995 [8], [9]. Beginning in 2000–2001, ivermectin was combined with albendazole in all 12 onchocerciasis endemic LGAs to treat co-endemic lymphatic filariasis (LF) [10]. In 2008, after 7–8 years of treatment, King et al conducted a survey to determine if LF transmission had been interrupted [11]. The King survey concluded that five of the original 12 co-endemic LF/oncho LGAs had interrupted LF transmission. MDA, however, was not approved by the Federal Ministry of Health to be stopped due to co-endemic onchocerciasis; accordingly the required post treatment surveillance for LF could not be launched. In an effort to ascertain whether onchocerciasis transmission had been interrupted after 17 years of MDA in these five LGAs, and to determine if all ivermectin-based MDA could be halted, the onchocerciasis study reported here was conducted in 2009. MDA has continued in these five LGAs through 2013, with ivermectin and albendazole (2010–2012) and then with ivermectin alone (2013).

The survey was structured to ascertain changes in microfilaria and nodule rates in villages for which baseline data were available, dating from the early 1990s, before ivermectin MDA was launched. In addition, we focused on two key criteria for halting MDA as outlined in the 2001 WHO document, “Certification of Elimination of Human Onchocerciasis: Criteria and Procedures,” [12]; 1) onchocerciasis infection rates in children of <0.1%; and 2) entomological infective (L3) larvae prevalence in the *S. damnosum* black fly vector <0.05%. These criteria were operationally adjusted to accommodate population challenges in assessing children by Lindblade in the Americas [13], [14], Higazi in Sudan [15] and Katabarwa, et al in Uganda [16]:

## Methods

### Ethics Statement

The State Ministries of Health for both Plateau and Nasarawa approved the surveys. The protocol was reviewed by the Emory Institutional Review Board (IRB) and considered as standard monitoring and evaluation, (e.g., deemed to be non-research under Federal Regulations 45 CFR Section 46.102(d)). Participants provided oral consent for their examinations, and parents who brought their young children for examination provided oral assent. Acceptance or denial of consent/assent was documented on survey forms. Persons >90 cm height and women who were not pregnant or lactating, were treated with ivermectin during MDA according to national program guidelines. Persons acting as human attractants for blackfly catches were told about the personal risks and community benefits of participation and given the option to opt-out of participation at any time without repercussions. Catchers were not compensated by the program.

### Study Area

**Figure 1:**
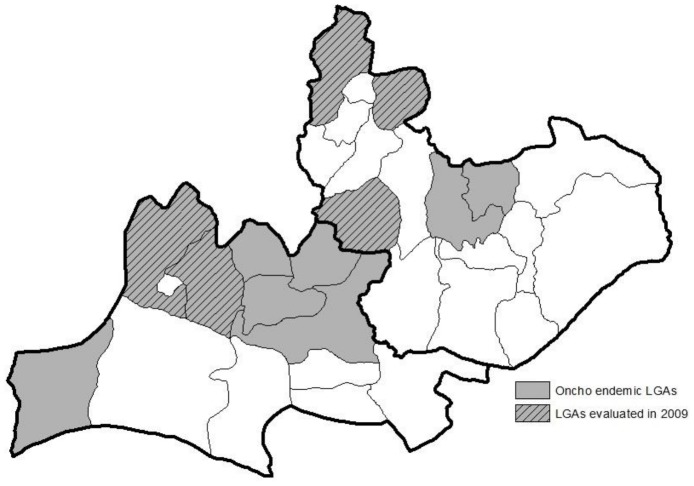
The evaluation of onchocerciasis transmission took place in five local government areas (shaded) in the states of Plateau and Nasarawa in north-central Nigeria.

Plateau and Nasarawa states are located in central Nigeria and have an estimated 4 million residents, most of whom live in agricultural villages. Plateau state was split into two (the eastern half forming Nasarawa state) in 1997. Administratively, the two states are divided into 30 LGAs: 17 in Plateau and 13 in Nasarawa. All 30 LGAs are LF endemic, and 12 of these also have meso-hyperendemic onchocerciasis (estimated nodule rates >20%). The five LF/onchocerciasis co-endemic LGAs where King et al determined that LF transmission had been eliminated are Karu, Kokona, Bokkos, Bassa and Jos East. These LGAs are located along the northwestern “oncho belt” of Plateau and Nasarawa states (Figure 1).

### 1991–1993 Baseline Data

Baseline data on nodule and/or mf prevalence were available for fourteen villages. Six of these were considered sentinel sites (sentinel villages) originally designated to be followed serially to measure the impact of the program. The other eight villages had baseline data for occasional ‘spot checks.’ The six sentinel villages consisted of two in Bassa and one in each of the other four LGAs (Table 1). The spot check villages consisted of three in Karu, two in Kokona, and one in each of the other three LGAs. A list of these villages is shown in Table 1, together with baseline data collected prior to launching ivermectin MDA, between 1991 and 1993.

**Table 1:** Onchocerciasis baseline evaluations (1991–1993) in fourteen villages (six sentinels and eight spot-check).

			Nodules			Microfilaria (mf)		
	LGA	Village	tested	positive	% positive	tested	positive	% positive
**Sentinel Villages**	Karu	Gurku	30	14	46.60%	30	12	40.00%
	Kokona	Arusu	na	na	na	30	13	43.33%
	Bassa	Bakin Kogi Lemoro	30	25	83.30%	na	na	na
	Bassa	Mafara	na	na	na	32	14	43.75%
	Bokkos	Kamwai	na	na	na	30	10	33.33%
	Jos East	Godong	27	2	7.40%	27	15	55.70%
		Subtotal	87	41	47.13%	149	64	42.95%
**Spot-check villages**	Karu	Sabon Ara	30	14	46.60%	na	na	na
	Karu	Takalafiya	30	12	40.00%	na	na	na
	Karu	Zhewun Yelwa	30	14	46.60%	30	12	40.00%
	Kokona	Ambasi	30	12	40.00%	na	na	na
	Kokona	Ninkoro	30	13	43.33%	na	na	na
	Bassa	Rimi	30	14	46.60%	na	na	na
	Bokkos	Richa	30	12	40.00%	na	na	na
	Jos East	Gada	31	5	16.13%	31	3	9.70%
		Subtotal	241	96	39.83%	61	15	24.59%
		Total	328	137	41.77%	210	79	37.62%

Baseline assessments were conducted on an average of 30 male adults, ≥20 years of age, and resident for at least five years, who were selected at random from among volunteers for evaluation. Sentinel sites were more likely (83%) to have baseline data that included mf prevalence than were spot check villages (25%).

Nodule assessments were conducted (by palpation) to find subdermal masses that met the characteristic clinical appearance of onchocercomas; rates were calculated as the total number of persons having any nodules divided by the total number examined. Skin snips were obtained from the left and right iliac crest using a field sterilizable punch biopsy instruments (corneoscleral punches). The skin snips (about 1–3 mg in weight) were incubated in normal saline and examined after 24 hours by microscopy (40×) for mf.

### Interval Treatment Coverage

The Plateau State Ministry of Health launched mass drug administration (MDA) with ivermectin in 1992, with the assistance of the River Blindness Foundation (RBF). The program treatment coverage goal was to reach at least 80% of the eligible population using community based distributors (CBDs) selected by the individual communities and trained by the RBF and Ministry staff. Ivermectin was provided to the CBD in the presence of the local chief at a time selected by the community leaders [8]. CBDs were then given 2–4 weeks to complete drug distribution and to report back to the Ministry of Health. Treatments were then verified by Ministry of Health staff.

Communities with the highest endemicity were targeted first: During the first year of treatment, 217,289 persons were treated in 636 communities, 72% of the treatment goal of 300,000. By 1994, treatments had increased to 448,521 in 939 communities, 75% of the treatment goal of 600,000. In 1995, the program achieved 87% of its treatment goal of 620,000 and has maintained >80% reported coverage ever since [9], [10]. In 1996, the strategy of MDA changed to the ‘Community Directed Treatment with Ivermectin’ (CDTI) strategy of the African Program for Onchocerciasis Control (APOC). In 2000, albendazole was added to the ivermectin MDA to treat lymphatic filariasis which was co-endemic with onchocerciasis. Treatment records do not allow disaggregating figures for the five LGAs in question, but overall reported coverage in the two state area remained >80% of the treatment eligible population for all years.

In 2003 a cluster coverage survey based on questionnaires provided independent treatment figures from treatment eligible persons drawn from a sample frame that included all 30 LGAs. The details of that survey were reported by Richards (2011) [10]. Questionnaire results showed an ivermectin/albendazole treatment coverage of 72.20% (95% CI 65.5–79.0) of the treatment eligible population in all 30 LGAs.

### 2009 Epidemiologic Evaluation

*Sentinel Village Surveys:* All 6 villages were surveyed at 12–14 months after the last round of MDA for prevalence of nodules, mf and onchocerciasis antibody (OV16). All eligible residents of the six sentinel villages (total population 13,512, range 692 to 3,840) were invited to participate and all those who arrived on the appointed day were enrolled. Name, age, years of residency, and last year of ivermectin treatment were all recorded. Nodule assessments were conducted by palpation in a manner similar to that used in the 1990s baseline surveys. If nodules were found, their locations were noted on a chart and the number recorded. Nodule rates were again calculated as the total number of persons having any nodules divided by the total number examined. The mf sampling methodology differed from the baseline assessments, because of concern about HIV and other viral infection risks when using field sterilization of corneoscleral punches. Therefore, in the 2009 surveys, disposable sterile needles and blades were used. After cleaning the skin with alcohol, the tip of a sterile needle was used to elevate 3–4 mm of skin over the right and left iliac crest. A sterile blade was then used to remove the skin at its base. The skin snip was then transferred to sterile saline solution in a 96 well plate and a second sample was taken from the left side using the same procedure. The blade and needle were then discarded. Skin snips were fixed in formalin after 24 hours incubation at room temperature and subsequently examined under a microscope (40×) for *O. volvulus* mf. Skin snips were obtained from the left and right iliac crest of adults (≥20 years) and children (3–12 years). Blood was collected by finger pricking. Blood samples were placed on Whatman No. 2 filter paper (Sigma-Aldrich, St. Louis, MO, USA), air dried, separated by sheets of paper, and stored in plastic bags in a cooler until they were returned to the laboratory in Jos and stored at 4**°**C. Blood samples were processed for IgG4 antibody against the OV16 recombinant antigen using a standard ELISA as described [13], [14]. ELISA testing was performed at The Carter Center laboratory in Jos, the capital of Plateau State.

### Expanded Surveys among Children in Spot Check Villages (Serological, mf and Nodules)

The 2001 WHO criteria for demonstration of interruption of transmission require an infection prevalence of <0.1% in children, with 95% confidence. This requires a sample size of at least 3,000 children. To reach this number, expanded surveys in children were conducted in the ‘spot-check’ villages and using the same methodology as above, children were examined for nodules and tested for mf (skin snip) and OV-16 (blood spot). Spot check villages were selected based on their having had high baseline (nodule) prevalence in the 1990s (see Results section). The results were combined with those from children in the sentinel villages.

### Entomological Survey

Capture sites were established at *Simulium* vector breeding points near the 6 sentinel villages. Villagers were asked to identify three to four persons that would serve as attractants on catch teams and two capture sites per village were established. Fly-catchers were from the sentinel villages and were supervised by the LGA ministry of health personnel. Fly catches took place during the peak black fly breeding season, from mid-June through August, 2009. Each site had four capture days per month, each consisting of hourly collections (50 minutes of catches and 10 minutes of rest) between 7:00am and 5:50pm. Flies were captured in tubes before they could bite, labeled, and preserved with 95% ethyl alcohol.

Flies were analyzed at The Carter Center lab in Jos. Analysis was done using O-150 polymerase chain reaction (PCR) and PCR products detected by ELISA to determine the presence of *O. volvulus* DNA [13], [14], [15]. Flies were grouped according to village into pools of 100 using positive controls. Fly heads and bodies were analyzed separately to determine if there was any infected flies (L1 or L2 in the bodies) or infective flies (L3 in the heads). If positives were found, the pool was run again for confirmation. L3 results were used to determine the seasonal transmission potential (STP), which is the theoretical number of L3s a person receives as the result of infective vector bites during the high transmission season.

### Statistical Analysis

Persons resident for <5 years, or children <5 years who had not resided all their lives in the community, were excluded in the final analysis. Mean comparison, chi square, and 95% confidence intervals for mf, nodules, and OV-16 were determined using STATA 11 (SataCorp LP, College Station, TX). A *P* value of <0.05 was considered statistically significant. Confidence intervals for blackfly analysis were calculated in PoolScreen 3.0 (University of Alabama, Birmingham, AL).

## Results

### Baseline Sentinel and Spot Check Village Surveys

Baseline results from the 1990s are shown in Table 1. Of the six sentinel villages surveyed, 3 had nodule prevalence data and 5 had mf data. Of the eight spot check villages, all 8 had nodule results and 2 had mf results. From 11 of the 14 villages, 328 persons were assessed for nodules and from 7 of the 14 villages, 210 persons were assessed for mf. The total nodule prevalence in the three sentinel villages was 47.10% (range 7.40–83.30%) and the total mf prevalence was 42.90% (range 33.30–45.60%) in the five sentinel villages with results. The total nodule prevalence in the eight spot check villages was 39.80% (range 16.10–46.60%) and the total mf prevalence in the two spot check villages was 24.60% (range 9.70–40.00%). Overall, mean nodule prevalence was 41.80% (range 7.40–83.30%) and the mean mf prevalence was 37.62% (range 9.70%–55.70%). Thirteen (93%) of the 14 villages were classified as being meso-hyperendemic (mf or nodule rates being >20%), the only exception being Gada (in Jos East) which was hypoendemic (nodule prevalence of 16%).

### 2009 Sentinel Village Survey

In the six sentinel villages, the total sample was 2,197 (739 adults and 1,458 children). Among the adults (aged ≥20), the mean age was 43 years and 43.12% were male (n = 329). The mean number of years resident in the village was 37 years, and 92.18% reported having taken ivermectin in the past. The majority of adults reported their occupation as “farmer” (68.62%), followed by “housewife” (19.09%), student (6.01%), “business” (2.94%), “other” (1.87%), civil servant (0.67%), and herdsman (0.40%).

Only 8 adults (4 male, 4 female) in the six sentinel villages had nodules, resulting in a prevalence of 1.08% (village range 0–4.00%); overall, this was a 97.60% reduction (p<0.001) from the 1991 baseline (97.41% amongst males alone as measured at baseline). Only two adults had positive skin snips, resulting in an mf prevalence of 0.27% (village range 0–1.82%), a 99.30% reduction (p<0.001) over baseline (Table 2). Twenty six adults had OV16 IgG4 antibody (3.52%, with village range 0–10.90%), which was 13 times the mf rate. Both individuals who were positive by skin snip were OV16 positive. Of the 8 adults with nodules, zero had mf in skin and 2 (25%) were OV16 positive.

**Table 2:** 2009 Sentinel village assessment for nodule, Mf, and OV16 prevalence among adults (persons aged ≥20 years).

		Nodule			Mf		OV16	
LGA	village	tested	Positive	% positive	positive	% positive	positive	% positive
Karu	Gurku	134	0	0.00%	0	0.00%	0	0.00%
Kokona	Arusu	108	1	0.90%	0	0.00%	5	4.63%
Bassa	Bakin Kogi Lemoro	160	0	0.00%	0	0.00%	2	1.25%
Bassa	Mafara	125	5	4.00%	0	0.00%	7	5.60%
Bokkos	Kamwai	110	2	1.80%	2	1.82%	12	10.91%
Jos East	Godong	102	0	0.00%	0	0.00%	0	0.00%
	**Total**	**739**	**8**	**1.08%**	**2**	**0.27%**	**26**	**3.52%**

Among children (3–12 years of age) and adults (≥20 years of age)								
		Nodule			Mf		OV16	
LGA	village	tested	positive	% positive	positive	% positive	positive	% positive
Karu	Gurku	354	0	0.00%	0	0.00%	0	0.00%
Kokona	Arusu	362	1	0.30%	0	0.00%	7	1.93%
Bassa	Bakin Kogi Lemoro	346	0	0.00%	0	0.00%	2	0.58%
Bassa	Mafara	410	5	1.20%	0	0.00%	8	1.95%
Bokkos	Kamwai	351	2	0.60%	2	0.57%	13	3.70%
Jos East	Godong	374	0	0.00%	0	0.00%	2	0.53%
	**Total**	**2197**	**8**	**0.36%**	**2**	**0.09%**	**32**	**1.46%**

Among children in sentinel villages, mean mf and OV16 rates were 0% and 0.41% respectively (6 OV16 positives, village range 0%–0.79%) (Table 3).

**Table 3:** 2009 Prevalence of Mf and OV16 among children, ages 3 to 12, in sentinel and spot check villages.

				Mf			OV16		
	LGA	village	tested	positive	% positive	upper 95% CI	positive	% positive	upper 95% CI
**Sentinel Villages**	Karu	Gurku	220	0	0.00%	1.66%	0	0.00%	1.66%
	Kokona	Arusu	254	0	0.00%	1.44%	2	0.79%	2.81%
	Bassa	Bakin Kogi Lemoro	186	0	0.00%	2.49%	0	0.00%	2.49%
	Bassa	Mafara	285	0	0.00%	1.29%	1	0.35%	1.94%
	Bokkos	Kamwai	241	0	0.00%	1.52%	1	0.41%	2.29%
	Jos East	Godong	272	0	0.00%	1.35%	2	0.74%	2.63%
		Sub total	1458	0	0.00%	0.26%	6	0.41%	0.92%
**Spot-check villages**	Karu	Sabon Ara	361	0	0.00%	1.02%	0	0.00%	1.02%
	Karu	Takalafiya	524	0	0.00%	0.70%	0	0.00%	0.70%
	Karu	Zhewun Yelwa	204	0	0.00%	1.79%	1	0.49%	2.70%
	Kokona	Ambasi	180	0	0.00%	2.03%	0	0.00%	2.03%
	Kokona	Ninkoro	171	0	0.00%	2.13%	0	0.00%	2.13%
	Bassa	Rimi	565	0	0.00%	0.62%	0	0.00%	0.62%
	Bokkos	Richa	505	0	0.00%	0.73%	0	0.00%	0.73%
	Jos East	Gada	483	0	0.00%	0.76%	0	0.00%	0.76%
		Sub total	2993	0	0.00%	0.12%	1	0.03%	0.18%
		Total	4451	0	0.00%	0.08%	7	0.16%	0.32%

The combined results for children and adults in the sentinel villages are shown on the lower panel of Table 2. The mean nodule prevalence was 0.36% (village range 0%–1.2%), the mean mf prevalence was 0.09% (range 0%–0.57%), and the mean OV16 prevalence was 1.46% (range 0%–3.70%).

### Expanded Surveys in Children in Sentinel and Spot Check Sites Combined

A total of 4,451 children ages 3 to 12 were examined in sentinel (1,458 children) and spot check (2,993 children) villages: 47.60% were female, the mean age was 8 years, and the mean number of years reported as resident was 8 years with 98.20% having been resident their entire lives. Among children who would have been eligible for ivermectin treatment during the previous year (i.e. ≥6 years), 90.20% reported having taken the drug. No positive skin snips or nodules were found (Table 3). In contrast, seven children were OV16 positive, resulting in a prevalence of 0.16% (village range 0–0.79%). OV16 rates were significantly higher in the sentinel villages (0.42%, range 0–0.79%) compared to the spot check villages (0.03%, range 0–0.49%) (p<0.002).

Adults in sentinel villages (Table 2) were more likely to be OV16 positive (p<0.001) and mf positive (p<0.01) than children (Table 1). There was no statistical difference in the prevalence of OV16 positive children by age group (p = 0.342), but older children, ages 10 to 12, had a seroprevalence that exceeded the 0.1% threshold (0.29%) while children under 10 were right on the 0.1% threshold (Table 4).

**Table 4:** Age specific prevalence of OV16 and skin microfilaria in children and adults in 5 LGAs of Plateau and Nasarawa States (combined sentinel villages and spot check villages).

age group	tested	OV16 positive	%OV16 positive	upper 95% CI	Mf positive	% Mf positive
3 to 5	1,131	1	0.09%	0.49%	0	0.00%
6 to 9	1,919	2	0.10%	0.38%	0	0.00%
10 to 12	1,401	4	0.29%	0.73%	0	0.00%
≥20	739	26	3.52%	5.11%	2	0.27%
total	5,190	33	0.64%	0.89%	2	0.04%

Of the seven OV16 positive children, only one, aged 10 (resident of Kamwai village, Bokkos LGA), had not been resident their entire life and had moved there at the age of 4. All but two of the OV16 positive children (ages 4 and 6) had received at least one round of ivermectin.

### Entomological Survey

The total number of black flies captured in the six sampled village was only 1,568. The highest number of flies caught in a single village was 568 in Kamwai and the lowest was 3 in Anacha. PCR runs on the head and body pools were negative for *O. volvulus* DNA. Since there were no positive heads, there were no infective larva and therefore the STP was 0.

## Discussion

Onchocerciasis and lymphatic filariasis (LF) have overlapping endemicity in many parts of Africa, and the ‘stop MDA’ decision for one is influenced by the transmission status for the other. This challenge was described by Katabarwa for the Wadalai focus of onchocerciasis in Nebbi District, Uganda [16]. In that case, ivermectin MDA for onchocerciasis could not be halted since ongoing transmission of LF required that MDA with ivermectin and albendazole continue. Katabarwa noted, “As elimination of onchocerciasis becomes more of a prospect in Africa, coordination of onchocerciasis and LF elimination efforts is essential in foci such as Wadelai where co-endemicity exists so that elimination of both diseases can be achieved in an integrated fashion, allowing similar interventions to be halted at the same time.” (Katabarwa et al (2012), page 6)

The situation in Plateau and Nasarawa States in Nigeria is the reverse side of the ‘oncho/LF stop MDA’ challenge. In 2009 King et al. showed that LF transmission had been interrupted in ten LGAs in the two state area, but the Federal Ministry of Health gave permission for stopping MDA only in five ‘LF only’ LGAs [11]. Permission was not granted to stop MDA in the five co-endemic oncho/LF LGAs until such time as onchocerciasis ‘stop MDA’ surveys could be completed effectively postponing post treatment surveillance for LF indefinitely.

There are two sets of epidemiological criteria for stopping onchocerciasis MDA currently in use: 1) The 2001 WHO Geneva criteria (used by the Onchocerciasis Elimination Program for the Americas [13], Sudan [15], and Uganda [16], which focus on demonstrating very low infection rates in young children as the primary support for claiming absence of recent transmission - very similar to the LF TAS survey approach [17]); and 2) the African Program for Onchocerciasis Control (APOC) criteria [18] based on work done in studies in Senegal and Mali [19] and in Kaduna, Nigeria [20]. In Senegal and Mali, Diawara, et al. used their observations to validate the ONCHOSIM model predicting transmission interruption would occur when overall village mf prevalence in a transmission zone fell below <5.0% in 100% of villages and <1.0% in 90% of villages [19]. Tekle et al (2012) reported that the annual ivermectin distribution program had achieved these mf thresholds in Kaduna state in Nigeria, a state that borders both Plateau and Nasarawa [20].

In our study we found epidemiological evidence suggesting that onchocerciasis transmission in the five oncho/LF LGAs of concern had been broken considering all the above criteria. Our surveys were designed to be most robust in demonstrating the 2001 WHO Geneva guideline requirement of very low infection rates in children. Skin snip prevalence was 0 in over 4000 children aged 3–12 years examined (upper 95% CI<0.01%) in our study. Community wide skin snip prevalence met APOC stop MDA requirements in all six sentinel villages, with total community (e.g., combined adults and children skin snip results) mf prevalences each being <1%, and no individual community being over 5%. Of note, APOC has subsequently conducted an independent evaluation of onchocerciasis throughout the two state endemic area and reported 100% negative skin snip findings from a more robust community sample [21]

Both WHO and APOC entomologic criteria for the onchocerciasis stop MDA decision seek infective (L3) rates in vectors <0.05% (with 95% confidence), requiring a sample size of at least 6000 flies. Unfortunately, our teams had difficulty capturing the required 6000 flies within the time frame and budgetary allowances of the study. We established 10 capture sites in four of the five LGAs of interest (Kokona, Bassa, Bokkos, and Jos East) with four capture days (7:00am and 5:50pm per day) per month during the black fly breeding season (mid-June through August) (e.g., 10 capture days per site). In other words, 100 person/days (and over 1000 person/hours) of human landing capture time was only sufficient to collect 1,568 *Simulium damnosum s.l.* flies, just 2.5% of the needed sample. To reach the WHO/APOC requirement, we would have had to increase our field activities to 400 person days (sixteen capture days per month), well beyond our logistical capacity. One recommendation from our study is that new approaches are needed for capturing *Simulium* vectors of onchocerciasis if these current entomological guidelines are to be met by the many programs seeking to stop MDA for onchocerciasis in this part of Nigeria. Operations research is needed to determine how to improve capture numbers without increasing the time required of human attractants. New black fly traps that are being developed may be an answer for doing this [22].

We found all 1,568 *S. damnosum s.l.* bodies and heads tested by PCR to be negative for *O. volvulus* DNA; as noted, this result is not sufficient to statistically exclude the WHO/APOC 0.05% infectivity threshold with 95% confidence. Despite this finding we are recommending that these results be used in support of a ‘stop MDA’ decision for the five LGAs. Important to note is that there was no evidence of any infection in the vector bodies, where earlier stage larvae (L1, L2) are found. Earlier (L1 and L2) stages in the flies are likely to be 3–5 times more frequent than L3s, and absence of any infection is as powerful as a ‘xenodiagnostic’ demonstrating the absence of mf in humans in these areas, and highly consistent with our skin snip results.

The evidence provided here (albeit entomologically incomplete) supports a conclusion that transmission of onchocerciasis had likely been broken in 2009. Given the fact that four more years of MDA have been provided since this study, and another MDA is planned for 2014, we think that interruption has been achieved now and that MDA could be stopped in 2015. Our results, however, apply only to the five oncho/LF co-endemic LGAs discussed. Another onchocerciasis survey is likely needed to make a decision on the remaining oncho/LF LGAs. Such a survey is would need to pay particular attention to collecting a sufficient number of black flies.

This paper and its companion by King et al. [11] provide an example of the complexities that encompass the stop MDA decisions in oncho/LF co-endemic areas. We favor the establishment of a Nigeria expert advisory committee to the FMOH, similar to the one the Ugandan Ministry of Health has established (the Ugandan Onchocerciasis Elimination Expert Advisory Committee) [16]. Such a committee would be very helpful in reviewing in detail the often incomplete, imperfect, or highly nuanced data sets, and so give best advice to the FMOH on where and when to halt interventions. As we have seen in this example, the onchocerciasis and LF programs should work closely together, perhaps through a single independent advisory review committee having combined oncho/LF responsibility to give a more rational approach to expediting the stop MDA process.

Our study gave us the opportunity to compare skin snip infection rates with the OV16 IgG4 ELISA. Among adults in the sentinel villages, OV16 antibody prevalence was 13 times greater than skin snip rates and 3.2 times greater than the nodule rates. All mf positive adults were OV16 positive, but the correlation with nodules (which were clinically onchocercomas but not parasitologically confirmed) was not as good. This is likely due to the well-recognized fact that as prevalence of onchocerciasis decreases, the specificity of clinical onchocercomas drops [23]. The fact that OV16 rates were low, 3.5% among long term resident adults in villages documented as being meso-hyperendemic in the 1990s, is evidence that the OV16 IgG4 response wanes over the years as transmission is broken and the adult *O. volvulus* parasites die and are not replenished.

The OV16 prevalence in children was higher than the skin snip prevalence, with no OV16 positive child having a positive snip. This could be due to the fact that their infections were below the sensitivity of a skin snip, or that their antibody response represented a recent exposure or a single sex infection, rather than a patent one. In the future, the use of PCR in skin snips from children who are OV16 positive might be considered as a better confirmatory test [24]. We also documented the classical increase of antibody positivity with increasing age. Given this, the general approach in the future should be to sample children aged under ten years for OV16 assessments [24], [16]. If we focus on children aged under ten, the OV16 antibody rate positivity rate was 0.10% (n = 3,050, 0.29% upper 95%CI). This raises another important issue, which is that the 2001 WHO guidelines do not distinguish ‘infection rates’ determined by nodule prevalence, mf prevalence, serological prevalence, or DEC patch test. Clearly more clarification is needed for the 1/1,000 infection benchmark. In this study we chose mf prevalence since this was the clearest representation of active infection of the three indices used (mf, nodule and OV16 antibody). The current data leave us asking ourselves ‘Is the OV16 antibody prevalence threshold (when used alone as an indicator of infection rates) of <0.1% too high of a standard for transmission interruption?’

Aside from the aforementioned weakness of the study in having insufficient numbers of black fly vectors to exclude the critical 0.05% infectivity threshold, there were at least three other weaknesses in this study. First, we compared the 1990s baseline nodule and mf rates determined in small samples of adult men with a larger follow up adult study that included adult women (57% of the sample). We also calculated 2009 community mf prevalence based on a sample that excluded the age groups 13–19 years. We do not believe either of these shortcomings dramatically altered the results or our conclusions, and we note that prevalence did not differ significantly between men and women in the 2009 sample. Second, the study was conducted in five LGAs selected based on the results of an earlier LF assessment by King. These LGAs may or may not be representative of the overall onchocerciasis transmission zone; it is uncertain because we do not know the subspecies of *S. damnosum* and its flight range. The worst case scenario would be a large transmission zone (e.g., far reaching flight range) that includes all the endemic LGAs in Plateau and Nasarawa as well as LGAs in neighboring Kaduna state. However, sentinel data (not presented here) from other onchocerciasis endemic LGAs in Plateau and Nasarawa show similar negative results (particularly in children), and published evidence from Kaduna suggests that transmission interruption is likely there as well [20]. Thus there is reason to suspect that transmission of onchocerciasis has been broadly interrupted by the MDA programs, even in the worst case scenario.

This study indicates the need, in onchocerciasis/LF co-endemic areas, to coordinate field assessments for the stop MDA assessments. If onchocerciasis assessments can be done together with LF transmission assessments surveys (TAS) [17] time and money can be saved in making a required joint programmatic decision to stop ivermectin based MDA. We urge that new tools and associated operations research be undertaken promptly to allow data that can be obtained concurrently and ideally from the same samples and age groups, to allow the stop MDA decision by technical committees with sufficient expertise in the epidemiological dynamics and accepted WHO guidelines for each disease.

## Supporting Information

Checklist S1 STROBE checklist.
(DOC)
